# Gratuities of Medical Men—An Appeal to the Legislature

**Published:** 1879-08-20

**Authors:** 


### *GRATUITIES OF MEDICAL MEN—AN APPEAL TO THE LEGISLATURE.*

---

Many years ago, Dr. R. C. Word, the Managing Editor of this Journal, at a meeting of the State Medical Association, introduced the following resolution, which was adopted.

*Resolved*, That this Association memorialize the Legislature of Georgia, to abolish the *Professional* tax upon physicians, and to urge the passage of an Act requiring the Inferior Court of each county to set apart such portion of the county tax as the Grand Jury shall recommend to purchase drugs for the use of the poor.

In accordance with this Resolution, Dr. Word, as chairman of the committee, drew up the following Memorial, which was duly presented to the Legislature, but failed of adoption for the want of backbone on the part of legislators who were afraid of being charged with class legislation, and who, as usual, were controlled by buncomb rather than by principles.

The Legislature being now in session we reproduce the document, hoping that the medical men who are members of that body will examine it, and frame and urge the passage of a bill embodying its suggestions, substituting the Ordinary for the Inferior Court to conform to present laws.

MEMORIAL.

The undersigned were appointed a Committee to bring this matter before your honorable body.

In performing this duty we beg respectfully to submit a few reasons for this appeal to the Law Making Power.

It is a fact, apparent to every observing mind, that medical men, far more than any other class of citizens, contribute gratuitously, their time, labor and money, to the relief of the indigent. And as it is the duty of the State authorities, and not of a particular class to bear the burden, these gratuitous services can be regarded in no other light than contributions to the State.

The per centage of population requiring these services is by no means inconsiderable. Taking the county of Floyd as an average county, observations have been made by which we are enabled, confidently, to assert that the amount thus donated by medical men in Georgia, in the single article of quinine can scarcely fall short of thirty thousand dollars per annum, with a strong probability that it largely exceeds that amount. If, in connection with this startling fact, we consider the value of other medicines contributed, with the time and labor, it is evident that the amount of this gratuitous outlay of physicians to the State is enormous. It is remarkable that the public, and especially the Legislature, has so little appreciation of the extent of these gratuitous labors of medical men. True it is that the Inferior Court, under existing laws, is required to provide for the poor, and the physician pays his part of the annual tax, levied for this purpose. But, either from defects of law, or gross neglect of the Courts, it is well known that the instances in which the poor are thus provided for are very few, and the procuring of medical services and drugs do not seem to be regarded as belonging to their list of duties. Indeed, except in cases of lunacy, or in extreme and rare instances of helplessness, where the subject has been entirely abandoned by his acquaintances, it is seldom that the Courts make any provision whatever. Yet, even in these extreme cases, deserted by all, the physician alone is expected, without compensation or thanks, to give, not only his personal attention, but his money, to the relief of the sufferer. At all seasons, in all kinds of weather, in the dark hours of night when others are asleep, the medical man passes from one scene of distress to another, bestowing his labor, risking his own health, and dispensing drugs to the indigent sick. To this course he is impelled by two powerful forces. The first and greatest is the demand of humanity, which to a conscientious man leaves often no alternative by which to escape the call. The second is the force of public sentiment, which will not tolerate in the physician that freedom of action which it allows to others. The merchant may refuse credit to whom he chooses. The druggist may decline to sell to an insolvent customer, and it is well, but the physician who exercises this liberty brings upon himself the severest censure, and consequent injury to his character and business.

To the many cases of casualty and death which occur in this fast age, a large proportion of which is among the poorer classes, the physician stands a ready servant, subject to every beck and call, and is expected and required to have in readiness all the appliances and material, at whatever cost—adapted to every emergency. By his promptness and benevolent agency he relieves large numbers, and oft times rescues them from impending death. When, under analogous circumstances, a party is snatched from a burning dwelling, or a watery grave, the individual who performs the deed is exalted into a hero. When a mariner rushes to the rescue of a distressed crew, he gains for himself laurels of praise and medals of honor. Not so with the physician. He is regarded as having discharged a mere common-place duty, and scarcely meets with a passing commendation. And such is the tyranny of custom and law, that if he refuses to respond to every call, he encounters the indignant frown of the community, and failing, from want of proper facilities or other cause, to adopt the most scientific treatment he becomes liable to prosecution and heavy damages. Although medical men, as a class, are proverbially benevolent and kind, and are ever ready to heed the call of suffering humanity; and while they claim and desire no special exemption from the moral responsibilities and duties incident to their noble profession, yet they feel that the public authorities can and ought to do more than is or has been done for the poor in this particular, and that they ought not to require the physicians' services nor his drugs without compensation; much less to heighten the infliction by imposing a specific tax. If it be urged that a physician's profession is his capital, and therefore ought to be taxed, we reply that it is taxed, and that heavily in the manner and for the reasons above stated. The calls of humanity and necessities peculiar to the practice of the medical profession; the exposure, irregular hours, impairment of health, encountering contagious maladies and raging epidemics, witnessing painful scenes, suffering and death, and the moral and legal responsibilities incurred; all bear heavily upon the practitioner, and can find no adequate compensation, even though the tax were removed, and the ordinary fees allowed in all cases.

We, therefore, respectfully petition your Honorable body to abolish the specific tax, and extend such relief to the poor and the medical profession in the matter under consideration, as wisdom and justice may suggest to the patriotic Representatives of a great State. And we feel well assured that such legislation so obviously necessary, so manifestly just and proper, and so highly called for by the growing philanthropy and benevolence of the present age, cannot fail to meet the sanction of a liberal and enlightened constituency.

